# Effect of Radial Extracorporeal Shock Wave Therapy on Pain Intensity, Functional Efficiency, and Postural Control Parameters in Patients with Chronic Low Back Pain: A Randomized Clinical Trial

**DOI:** 10.3390/jcm9020568

**Published:** 2020-02-19

**Authors:** Karolina Walewicz, Jakub Taradaj, Maciej Dobrzyński, Mirosław Sopel, Mateusz Kowal, Kuba Ptaszkowski, Robert Dymarek

**Affiliations:** 1 Faculty of Physiotherapy, Opole Medical School, 45-060 Opole, Poland; karolina.w101@wp.pl; 2 Institute of Physiotherapy and Health Sciences, Academy of Physical Education, 40-065 Katowice, Poland; j.taradaj@awf.katowice.pl; 3 College of Rehabilitation Sciences, University of Manitoba, Winnipeg, MB R3E 0T6, Canada; 4 Department of Conservative Dentistry and Pedodontics, Wroclaw Medical University, 50-425 Wroclaw, Poland; maciej.dobrzynski@umed.wroc.pl; 5 Department of Nervous System Diseases, Wroclaw Medical University, 61-618 Wroclaw, Poland; mirek.sopel@gmail.com; 6 Department of Physiotherapy, Wroclaw Medical University, 51-355 Wroclaw, Poland; mateusz.kowal@umed.wroc.pl (M.K.); kptaszkowski@gmail.com (K.P.)

**Keywords:** extracorporeal shock wave therapy, orthopedic diseases, low back pain, functional ability, pain level, postural stability, rehabilitation

## Abstract

Low back pain (LBP) is the leading cause of disability worldwide, placing a significant economic burden on healthcare systems. Radial extracorporeal shock wave therapy (rESWT) is useful in the rehabilitation of orthopedic diseases; however, there is still limited evidence for patients with LBP. The aim of this study was to assess the effect of rESWT on pain level, functional efficiency, and parameters of postural control in patients with LBP. Participants were randomized into group A (*n* = 20) treated with rESWT and group B (*n* = 20) treated with sham rESWT (placebo). Both groups received conventional physiotherapy, including core stability exercises. The following tests were performed: the Laitinen Pain Scale (LPS), the Roland–Morris Questionnaire (RMQ), the original Schober Test (OST), and a stabilometric platform for the assessment of postural sway, including total sway path (TSP). We found that the analgesic effect was higher after rESWT, especially in the follow-up’s (*p* < 0.05). Similar results were found for functional efficiency and range of motion (*p* < 0.05). The improved posture stability in placebo group B was not statistically significant (*p* > 0.05). The stabilometric parameters in group A were still gradually improved and statistically significant, even in follow-ups (*p* < 0.05). In conclusion, the rESWT had a significant effect on the reduction of pain and the improvement of functional condition compared to a conventional physiotherapy program. Also, rESWT with core stability exercises led to significant improvements in postural sway compared with conventional physiotherapy in patients with LBP.

## 1. Introduction

Low back pain (LBP) is the leading cause of disability worldwide, having a substantial effect on patients’ quality of life (QOL) and placing a significant economic burden on healthcare systems. In the majority of cases, treatment of chronic LBP begins with pharmacological conservative management, such as opioid medications, lumbar epidural steroid injections (LESI), muscle relaxants, or nonsteroidal anti-inflammatory drugs (NSAIDs). The total cost and utilization of the maximum nonoperative therapies (MNTs) for LBP are still rising [1].

In 2018, the Lancet LBP Working Group identified a global problem of LBP mismanagement [2,3,4]. The phenomenon of unnecessary care in both high- and low-income settings was documented, whereby patients receive health services that are discordant with international guidelines. The strong evidence was presented that unnecessary care, including pain medications, spinal imaging tests, spinal injections, and surgical operations, is hazardous for most patients with LBP.

Therefore, searching for modern, low-cost, and effective new therapies is strongly recommended. The use of various nonpharmacological supportive and conservative methods in LBP management is well-known, including exercise training [5,6], manual therapies [6,7], or myofascial techniques [8]. However, effective physical agents are also needed [9,10,11].

One of the promising physical methods for the treatment of musculoskeletal pain syndromes is an extracorporeal shock wave therapy (ESWT). From the biophysical point of view, shock waves are defined as a sequence of high energy mechanical pulses generating short-term pressure changes during their propagation [12]. In general, there are two types of ESW, which differ in terms of the method and the range of acoustic energy propagation [13].

**Figure 1:**
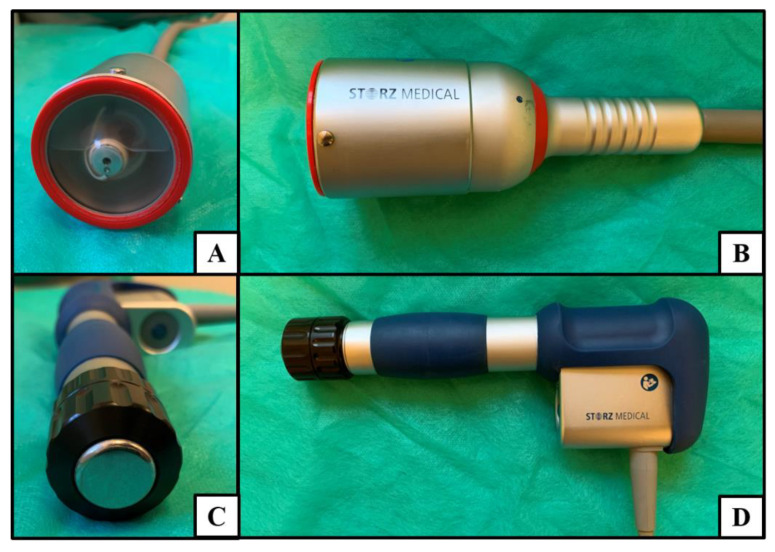
Extracorporeal shock wave applicators: (**A**) front and (**B**) lateral view of focused extracorporeal shock wave therapy (fESWT) (electromagnetic type—EM); (**C**) front and (**D**) lateral view of radial extracorporeal shock wave therapy (rESWT) (pneumatic type—PN).

Focused ESWT (fESWT) is generated by electromagnetic, electrohydraulic, or piezoelectric methods. The energy of fESWT increases rapidly < 10 ns, reaching high peak values of 10–100 MPa (absorbency up to 12 cm). The fESWT beam has a concentrated shape and so-called focal point, which is the highest energy density in a relatively small area. In turn, the radial ESWT (rESWT) is generated by the pneumatic method and generates slowly increasing pressure up to 5 µs, reaching the level of 0.1–1.0 MPa (absorbency up to 3 cm) with dispersed beam shape [14]. It should be noticed that both types of ESWT also differ in the size and shape of the applicators’ heads (Figure 1).

The ESWT also differs according to the level of energy delivered during a single pulse—energy flux density (EFD). Low energy doses for the non-invasive purposes are usually in the range of 0.01–0.12 mJ/mm^2^, while high doses reach even 0.2–0.4 mJ/mm^2^. Usually, the amount of 500–4000 pulses is applied during therapy, and the frequency of pulses oscillates between 1–10 Hz [15].

Therapeutic mechanisms of the ESWT have been documented through in vitro trials and animal experiments. Promising reports from the field of basic research concerning the search for reliable therapeutic mechanisms after the application of the ESWT cause that doctors and physiotherapists increasingly use this physical agent in their clinical practice. However, for spinal pain syndromes, the ESWT is not yet widely used and is still a novelty that requires scientific verification.

Therefore, the primary purpose of this study was to evaluate the effects of rESWT on pain intensity and functional efficiency in patients with chronic LBP. The secondary purpose was to assess the effect of rESWT on biomechanical parameters of postural control parameters in LBP patients.

## 2. Material and Methods

### 2.1. Study Design

The research was performed from April 2018 to November 2018 at an outpatient clinic of the Opole Medical School, Poland. The study was approved by the independent Bioethics Committee of the Wroclaw Medical University, Poland (approval no. KB-75/2017). All participants gave their written informed consent to participate in the study, which was carried out in accordance with the tenets of the Declaration of Helsinki and Good Clinical Practice guidelines [16]. The whole study was reported according to the requirements of the Consolidated Standards of Reporting Trials (CONSORT) statement [17]. This randomized clinical trial (RCT) was prospectively registered in the Australian New Zealand Clinical Trial Registry Platform (no. ACTRN12618000593235) supervised by the World Health Organization (WHO).

### 2.2. Participants

Patients with discopathy of the L5–S1 spine segment with chronic pain lasting more than three months were included in the study. The diagnosis was based on magnetic resonance imaging (MRI) that determined the advancement of degenerative and inflammatory changes of the lumbar region (>Modic III°). The exclusion criteria were acute LBP; sciatica episodes; degenerative changes of the cervical or thoracic region (individuals with lesions I° and II° according to Modic classification were not excluded from the study; only degeneration III° was a basis for exclusion); past fractures of the bone structures of the spine; cancer; vertebra forward dislocation; rheumatoid arthritis and ankylosing spondylitis; cauda equina syndrome; pregnancy; acute and chronic cardiovascular diseases; arrhythmia and pacemaker; metal implants; dermatological conditions in the area of the rESWT application; sensory deficits; psychiatric disorders; immunological diseases; infections; chronic drug use; problems with the balance system; and central nervous system diseases.

**Figure 2:**
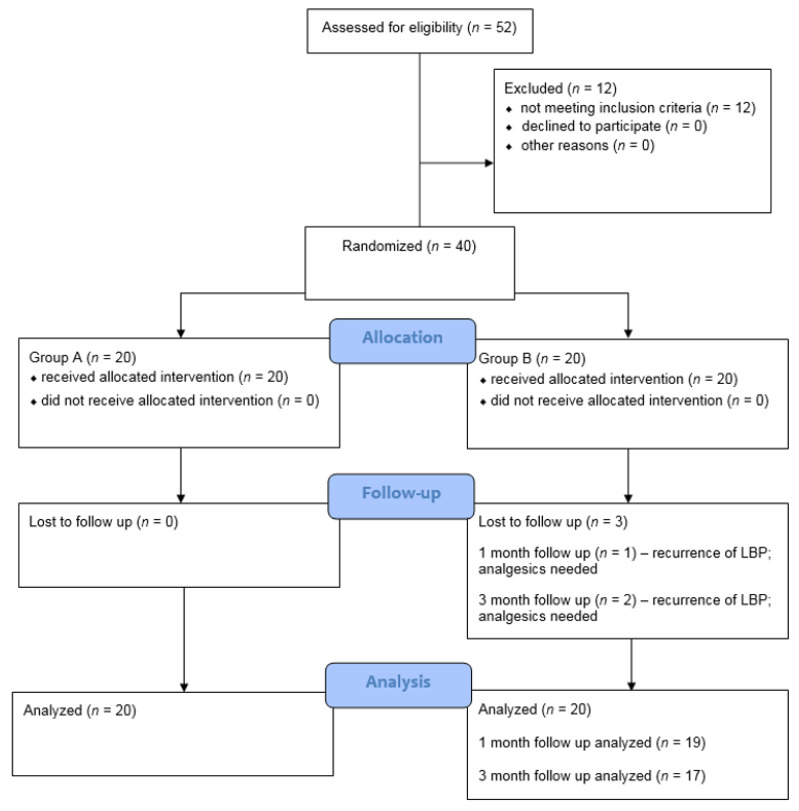
The CONSORT 2010 flow chart of patients in the study.

At the final stage, 37 out of 40 patients were evaluated since three participants dropped out in the follow-up observation due to the recurrence of LBP symptoms in group B and the necessity to use analgesics (Figure 2). There was no withdrawal due to ESW-related side effects. The subjects’ demographics and clinical characteristics are summarized in Table 1. There were no statistically significant differences between the demographic and other factors, such as pain level, functional condition, range of motion (ROM), and postural stability parameters, between the two groups.

### 2.3. Sample Size

The sample size of this research was based on group differences in the primary outcomes (means and standard deviations of pain feelings), which were estimated to 20 participants. In calculating, we allowed for a 20% loss of follow-up and historical information from our unit, as 45% of patients who were offered conservative (standard physiotherapy) management for this condition opted for extracorporeal shock wave therapy (ESWT) within six months. The sample size analysis was performed using Statistica 13.1 (TIBCO Software Inc., Greenwood Village, CO, USA).

### 2.4. Randomization and Blinding

This study was designed as a single-blind randomized controlled study. After baseline assessments, the participants were randomly assigned to an rESWT and core stability exercises or a sham rESWT and core stability exercises group. The individuals receiving the treatment were blinded. A computer-generated list of random numbers was used and concealed from the researchers enrolling and assessing the participants. The outcome assessors and data analysts were kept blinded to the allocation.

### 2.5. Interventions

The treatment protocol in group A included core stability training (45 min, once a day, five days a week from Monday to Friday) with myofascial relaxation of the erector spinae muscle, activation of the lumbo-pelvic-hip complex and deep core muscles training, exercises stimulating breathing, dynamic postural exercises, and treatment sessions with rESWT (2.5 bars, 2000 pulses, 5 Hz, 7 min) using the Pro-Shock Waves pneumatic device (Cosmogamma, Jakarta, Indonesia). The rESWT procedures were performed twice a week (on Monday and Thursday) for a period of five weeks (total of 10 procedures).

Participants in group B were treated with the same core stability training as the previous group and additionally with the sham rESWT. The shock wave therapy was identical to that of group A (the sham stimulation was voided of biologically active components by applying a unique polyethylene applicator cap, which absorbed energy and limited its propagation to the patient tissues) with the same sound signals of the pneumatic applicator head during the procedure and the same technical parameters as in the active shock wave procedures. Both the rESWT sessions and core stability exercises were performed by two certified physiotherapists with over three years of relevant experience, and the sessions were performed according to the literature [18].

### 2.6. Outcome Measurements

The clinical examinations were performed by an independent observer who had no information about the treatment protocol and were conducted during clinic visits at baseline and after treatment (at the end of 5 weeks’ active therapy; 1- and 3-month follow-up). The outcome measurements were assessed using the Laitinen Pain Scale (LPS) for the level of pain, the Roland–Morris Questionnaire (RMQ) for the degree of functional condition, and the original Schober Test (OST) for range of motion (ROM), as well as stabilometric platform for postural sway parameters.

The LPS was used to assess subjective pain level, including its intensity and frequency, as well as information about analgesic pharmacotherapy used, and pain-related reduced functional mobility. The total score for these four domains ranges between 0–16 points, while a lower score indicates a better state of the patient [19].

The RMQ was used to evaluate the degree of disability and state of functional condition. This questionnaire contains 24 items scored of 0 points (answer “no”) or 1 point (answer “yes”), indicating lack of disability (0–3 points) or minimal (4–10); moderate (11–17) and severe disability (18–24) [20].

The OST was used to measure the ROM of the lumbar spine. The assessment was performed at the level of L5, with two points marked 5 cm below and 10 cm above within a total distance of 15 cm. The patient was asked to flex the trunk with the possible touch of the toes while keeping the knees straight [21].

The stabilometric platform CQ Stab2P (CQ Elektronik System, Czernica, Poland) was used to analyze a postural stability parameter, including a total sway path (TSP). A single test lasted 30 s and was conducted in both groups, with eyes open and closed. A research utility of a stabilometric platform for biomechanical assessment of postural control in patients with LBP is well investigated [22,23,24,25].

### 2.7. Statistical Analysis

The chi-square test was used to compare the sex between the two groups. All quantitative variables were tested using the Shapiro–Wilk test to determine the distribution type. Due to the lack of a normal distribution and the low sample size, the analyses were performed using non-parametric tests. Mann–Whitney U test was used to compare the age, height, and body weight, duration of symptoms, Modic class, LPS, RMQ, OST, and TSP between both groups before the treatment. Mann–Whitney U test was also used to compare outcomes between the two groups after the treatment. The comparisons of results obtained before and after the ESWT and at 1- and 3-month follow-up were carried out using non-parametric repeated-measures analysis of variance. Statistical calculations were performed with STATA 15 (StataCorp LLC, Lakeway Drive College Station, TX, USA) with a significance level of *p* < 0.05.

## 3. Results

**Figure 3:**
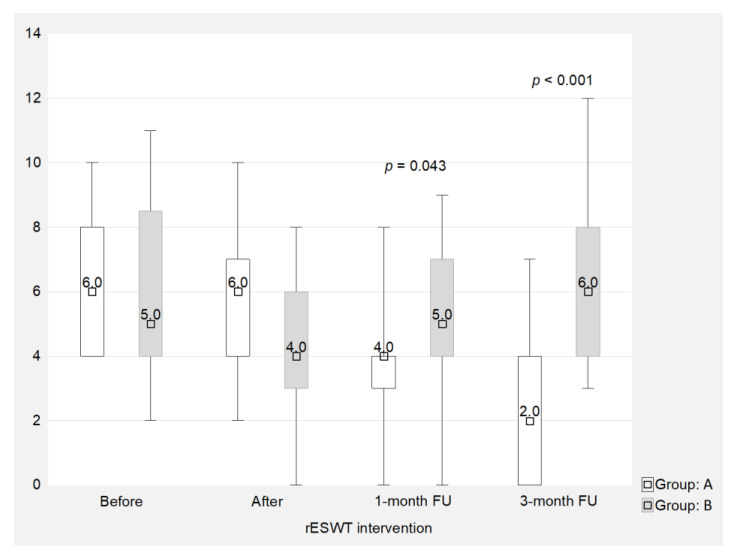
Comparison of pain outcomes (LPS) between two groups (points). LPS: Laitinen Pain Scale.

Both groups demonstrated a decrease in pain intensity throughout the treatments. However, the analgesic effect was more effective after rESWT, especially in the follow-up period There were statistically significant differences between the groups after 1 month (4 points in group A vs. 5 in group B; *p* = 0.043) and 3 months from the end of therapy (2 points in group A vs. 6 in group B; *p* < 0.001), respectively (Figure 3).

**Figure 4:**
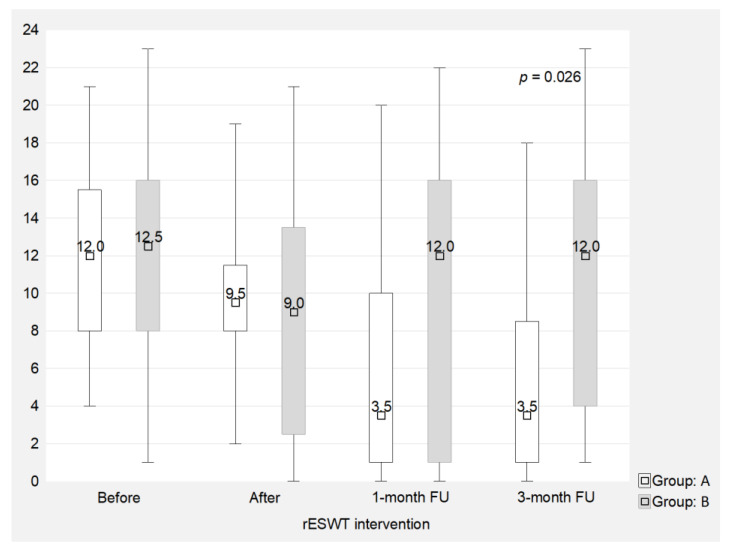
Comparison of functional outcomes (RMQ) between two groups (points). RMQ: Roland–Morris Questionnaire.

**Figure 5:**
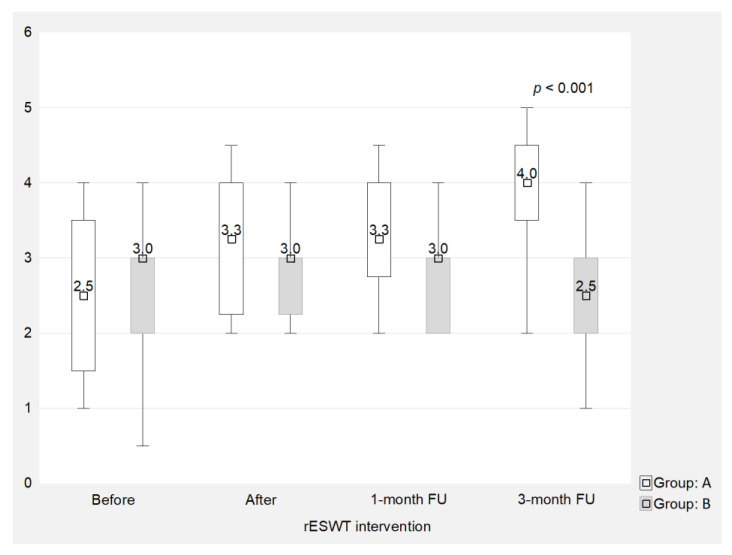
Comparison of the mobility outcomes (OST) between two groups (cm). OST: original Schober Test.

The situation was very similar for the functional efficiency assessment using the RMQ (Figure 4) and the ROM assessment in the lumbar segment using the OST (Figure 5). The stronger therapeutic effect after rESWT accelerated significantly in the follow-up observations (3.5 vs. 12 RMQ points, and an OST score of 4 vs. 2.5 cm in favor of rESWT compared to the sham procedure at three months after the end of the treatment program).

The procedures undertaken in the comparison groups led to improved posture stability in the patients. These positive changes in group B were mainly in short-term observations (after 5 weeks’ therapy). This finding indicated that all these positive changes in the results (though not statistically significant) were very unstable and only observed in a short-term period. Interestingly, it was also noted that after rESWT (group A) in the follow-up analysis (1 and 3 months after therapy without continuing core stability exercises), the parameters remained improved at a statistically significant level (Table 2 and Table 3).

## 4. Discussion

To the best of our knowledge, the present study is currently the first worldwide RCT to evaluate the effectiveness of rESWT with a homogeneous population of patients with chronic LBP in the field of posture stability parameter analysis. Moreover, the previous studies used only subjective measurement tools (questionnaires, surveys, scales) for the assessment of clinical parameters. Novel elements also include the evaluation of the early and follow-up findings and attempts to determine the placebo effect of ESWT using a placebo-controlled study protocol.

Our study demonstrated that rESWT interventions led to significant analgesic effect, which was observed, especially in the follow-up observations. This fact might be caused by the long-term therapeutic effect of the rESWT wave in LBP patients, which has a beneficial clinical effect in terms of a longer period of remission of the spinal pain symptoms. Similarly, clinically relevant results were demonstrated for functional efficiency (LPS and RMQ) and spinal mobility (ROM). The improved posture stability during the biomechanical assessment of postural sway was shown after rESWT sessions compared to the placebo group that received the sham-rESWT intervention and conventional physiotherapy program, even in follow-up analyses. Obtaining positive results in this scope indicated a beneficial effect of rESWT treatment sessions on postural control and overall body stability.

The current systematic review of the literature on the efficiency of ESWT in LBP patients shows that, despite increasing popularity and novelty of this method, there are only a small number of RCTs, especially for rESWT. Moreover, many of these studies do not meet the appropriate methodological criteria of evidence-based medicine. Ultimately, it makes very difficult to analyze the clinical usefulness of these common procedures in an objective manner.

Tomska et al. [26] presented a study, comparing the effectiveness of deep electromagnetic stimulation (DEMS) and rESWT in the subjective assessment of patients suffering from LBP. The study enrolled 73 patients who were divided into group A (*n* = 25), which underwent 6 sessions of rESWT (1.5–2.5 bars, 2000 pulses, 5–10 Hz), and group B (*n* = 27) treated with 10 sessions of DEMS (50 Hz, 2.5 T, 10 min). Both groups demonstrated a significant reduction of pain (*p* = 0.03), with no statistically significant differences between these groups (*p* = 0.227). However, methodological limitations of this study should be emphasized: no blinding (subjects, therapists, or assessors), no adequate follow-up, no intention-to-treat analysis. Unfortunately, this study was scored only 3/10 points according to the PEDro (Physiotherapy Evidence Database) scale due to its limitations.

Han et al. [27] conducted a study with 30 patients with chronic LBP who were divided into a conservative physical therapy group—CPT (*n* = 15; hot packs, ultrasound, transcutaneous electrical nerve stimulation—TENS) and an ESWT group (*n* = 15; 1.5–3 bars, 1000 pulses, 2.5 Hz). Pain intensity was assessed using a visual analog scale (VAS), level of disability was assessed using the Oswestry Disability Index (ODI), and degree of depression was measured using the Beck Depression Index (BDI). It was concluded that ESWT is an effective intervention for the treatment of pain, disability, and depression in chronic LBP patients because intergroup comparisons revealed that these decreases in the VAS, ODI, and BDI scores were significantly larger in the ESWT group than in the CPT group. This study also received only 3/10 points on the PEDro scale.

Another study by Lee et al. [28] included 28 patients with chronic LBP persisted for 12 weeks or longer. Patients were divided into an ESWT group (*n* = 13, 2.5 bars, 2000 pulses, 5 Hz) and a CPT group (*n* = 15, hot packs, ultrasound, and TENS). Both groups took part in an exercise program comprised of Williams and McKenzie’s exercises (30-min sessions, twice a week, for six weeks.) The Williams exercises were composed of a posterior pelvic tilt (10 sec/1 set, 3 sets), followed by sit-ups (10 times/1 set, 3 sets) and a knee-to-chest exercise (10 sec/1 set, 3 sets). The McKenzie’s exercises involved bending the trunk back while supporting it with both elbows in a prone position (trunk extension) (20 sec/1 set, 3 sets), followed by bending the trunk back while supporting it with both hands, with the elbow extended in a prone position (10 sec/1 set, 3 sets), and then bending the trunk back in a standing position (10 sec/1 set, 3 sets). Korean researchers concluded that the exercise program combined with ESWT relieved chronic LBP more than the exercise program combined with the CPT. Similar limitations as above were found in this study—3/10 points in PEDro classification.

The present study has also some potential methodological limitations, which need to be mentioned. First of all, the collected material should be verified by other research centers to confirm or overthrow the results obtained in this study. In the future, the performance of rESWT should be verified using more precise measuring tools (e.g., surface electromyography, isokinetic systems, or 3D gait assessment). It would also be interesting to confront the ESWT itself with other physical procedures commonly used in everyday clinical practice among LBP patients (e.g., electrical therapies, including TENS or high voltage electrical stimulation—HVES, or therapeutic ultrasound). A small number of patients in both groups is also a limitation of this study; nevertheless, the pilot character of this study can be justified with its novelty. It should be pointed out that the future well-designed studies with a larger sample are still in demand to verify clinical utility and therapeutic efficiency of rESWT in patients with LBP.

## 5. Conclusions

The rESWT had a significant influence on the reduction of pain and the improvement of patients’ general functional condition in relation to a conventional physiotherapy program. These favorable effects were observed, especially in the long-term period. The rESWT with core stability exercises led to significant improvements in the postural sway in patients with LBP compared with standard core stability training.

## Figures and Tables

**Table 1:** Characteristics of the participants included in the study.

	Group	Parameters			
		*n*	$\bar{x}$	SD	*p*-Value
Age (years)	A	20	51.1	8.4	0.105
	B	20	55.8	9.3	
Height (cm)	A	20	165.7	7.8	0.465
	B	20	165.0	7.7	
Body weight (kg)	A	20	77.2	17.3	0.394
	B	20	80.4	15.2	
Duration of symptoms (years)	A	20	9.8	5.1	0.725
	B	20	9.0	4.1	
Modic classification (^o^)	A	20	3.1	0.2	0.797
	B	20	3.1	0.3	
Sex (*n*/%)	A	Women *n* = 14 (70%) Men *n* = 6 (30%)			0.723
	B	Women *n* = 15 (75%) Men *n* = 5 (25%)			

Abbreviations: *n*, number of patients; $\bar{x}$ , average; SD, standard deviation.

**Table 2:** Results of the total sway path (TSP) with open eyes in both groups (mm).

**Group A**					
	***n***	$\bar{x}$	**SD**	*p*-Value (Main Effect) *	*p*-Value (Multiple Comparisons) **
Before	20	236	52.52	***p* = 0.024**	Before: After *p* = 0.257 Before: 1-month FU ***p* = 0.008** Before: 3-month FU ***p* = 0.003** After: 1-month FU ***p* = 0.041** After: 3-month FU ***p* = 0.032** 1-month: 3-month *p* = 0.837
After	20	224	44.28		
1-month FU	20	199	32		
3-month FU	20	195	34.24		
**Group B**					
	***n***	$\bar{x}$	**SD**	*p*-Value (Main Effect) *	*p*-Value (Multiple Comparisons) **
Before	20	232	62.22	*p* = 0.334	Before: After *p* = 0.512 Before: 1-month FU *p* = 0.839 Before: 3-month FU *p* = 0.897 After: 1-month FU *p* = 0.730 After: 3-month FU *p* = 0.722 1-month FU: 3-month FU *p* = 0.925
After	20	223	57.22		
1-month FU	19	233	60		
3-month FU	17	234	64.76		

Abbreviations: n, number of patients; $\bar{x}$ , average; SD, standard deviation; FU, follow-up. * Friedman test; ** Dunn test. **Note:** *p*-values with statistical significance are presented in bold.

**Table 3:** Results of the total sway path (TSP) with closed eyes in both groups (mm).

**Group A**					
	***n***	$\bar{x}$	**SD**	*p*-Value (Main Effect) *	*p*-Value (Multiple Comparisons) **
Before	20	318	57.95	***p* = 0.041**	Before: After *p* = 0.310 Before: 1-month FU ***p* = 0.018** Before: 3-month FU ***p* = 0.026** After: 1-month FU ***p* = 0.048** After: 3-month FU *p* = 0.079 1-month FU: 3-month FU *p* = 0.637
After	20	312	56.44		
1-month FU	20	302	58.45		
3-month FU	20	309	50.24		
**Group B**					
	***n***	$\bar{x}$	**SD**	*p*-Value (Main Effect) *	*p*-Value (Multiple Comparisons) **
Before	20	320	73.10	*p* = 0.424	Before: After *p* = 0.312 Before: 1-month FU *p* = 0.479 Before: 3-month FU *p* = 0.510 After: 1-month FU *p* = 0.880 After: 3-month FU *p* = 0.712 1-month FU: 3-month FU *p* = 0.725
After	20	315	65.50		
1-month FU	19	316	66.24		
3-month FU	17	319	70.76		

Abbreviations: *n*, number of patients; $\bar{x}$ , average; SD, standard deviation; FU, follow-up. * Friedman test; ** Dunn test. **Note:** *p*-values with statistical significance are presented in bold.
