# Correction: Walewicz et al. Effect of Radial Extracorporeal Shock Wave Therapy on Pain Intensity, Functional Efficiency, and Postural Control Parameters in Patients with Chronic Low Back Pain: A Randomized Clinical Trial. *J. Clin. Med.* 2020, *9*, 568

**DOI:** 10.3390/jcm15114122

**Published:** 2026-05-27

**Authors:** Karolina Walewicz, Jakub Taradaj, Maciej Dobrzyński, Mirosław Sopel, Mateusz Kowal, Kuba Ptaszkowski, Robert Dymarek

**Affiliations:** 1 Faculty of Physiotherapy, Opole Medical School, 45-060 Opole, Poland; 2 Institute of Physiotherapy and Health Sciences, Academy of Physical Education, 40-065 Katowice, Poland; 3 College of Rehabilitation Sciences, University of Manitoba, Winnipeg, MB R3E 0T6, Canada; 4 Department of Conservative Dentistry and Pedodontics, Wroclaw Medical University, 50-425 Wroclaw, Poland; 5 Department of Nervous System Diseases, Wroclaw Medical University, 61-618 Wroclaw, Poland; 6 Department of Physiotherapy, Wroclaw Medical University, 51-355 Wroclaw, Poland

## Error in Figure

In the original publication [1], there was a mistake in “**Figure 3.** Radial extracorporeal shock wave applicators: (**A**) the standard rESWT applicator and (**B**) sham-rESWT applicator with polyethylene cap to provide placebo interventions” as published. The image included in the published version did not correspond to the device system used during the study and was inadvertently included during manuscript preparation. The rESWT intervention in the study was performed using a Pro-Shock Waves pneumatic device (Cosmogamma, Jakarta, Indonesia) with predefined and consistently applied parameters (2000 impulses, 2.5 bar air pressure, 5 Hz frequency). For clarity and to avoid potential confusion, Figure 3 has been removed from the article along with its reference in the text. The following figures—Figure 4, Figure 5 and Figure 6—are renamed to Figure 3, Figure 4 and Figure 5, respectively. 

## Text Correction

In Section 2.5. Interventions, paragraph 1 was corrected from “The treatment protocol in group A included core stability training (45 min, once a day, five days a week from Monday to Friday) with myofascial relaxation of the erector spinae muscle, activation of the lumbo-pelvic-hip complex and deep core muscles training, exercises stimulating breathing, dynamic postural exercises, and treatment sessions with rESWT (2.5 bars, 2000 pulses, 5 Hz, 7 min) using the ESW CELLACTOR® SC1 (Storz Medical AG, Tägerwilen, Switzerland). The rESWT procedures were performed twice a week (on Monday and Thursday) for a period of five weeks (total of 10 procedures).” to

The treatment protocol in group A included core stability training (45 min, once a day, five days a week from Monday to Friday) with myofascial relaxation of the erector spinae muscle, activation of the lumbo-pelvic-hip complex and deep core muscles training, exercises stimulating breathing, dynamic postural exercises, and treatment sessions with rESWT (2.5 bars, 2000 pulses, 5 Hz, 7 min) using the Pro-Shock Waves pneumatic device (Cosmogamma, Jakarta, Indonesia). The rESWT procedures were performed twice a week (on Monday and Thursday) for a period of five weeks (total of 10 procedures).

The authors state that the scientific conclusions are unaffected. This correction was approved by the Academic Editor. The original publication has also been updated.
